# Qualität der Cochleaimplantat-Rehabilitation unter COVID-19-Bedingungen

**DOI:** 10.1007/s00106-020-00922-0

**Published:** 2020-09-02

**Authors:** A. Aschendorff, S. Arndt, S. Kröger, T. Wesarg, M. C. Ketterer, P. Kirchem, S. Pixner, F. Hassepaß, R. Beck

**Affiliations:** Klinik für Hals‑, Nasen- und Ohrenheilkunde, Albert-Ludwigs-Universität Freiburg im Breisgau, Killianstr. 5, 79106 Freiburg, Deutschland

**Keywords:** Sprachtherapie, Nachsorge, Severe acute respiratory syndrome coronavirus 2, Qualitätssicherung, Versorgungsstandard, Speech therapy, Aftercare, Severe acute respiratory syndrome coronavirus 2, Quality assurance, Standard of care

## Abstract

**Hintergrund:**

Die Rehabilitation nach CI(Cochleaimplantat)-Operation erfolgt leitliniengerecht durch eine multimodale Therapie, technische Anpassungen des Sprachprozessors und medizinische Nachsorge. Zu Zeiten der Corona-Pandemie wurde für die Patienten der Zugang zur auditorischen Rehabilitation verzögert oder erschwert. Die neuen Hygienemaßnahmen durch die SARS-Cov-2-Pandemie verändern auch die medizinische Nachsorge und Rehabilitation nach CI. Ziel der Untersuchung war es, die Qualität der Rehabilitation unter Corona-Bedingungen zu evaluieren.

**Material und Methoden:**

Wir führten eine anonyme Befragung erwachsener Rehabilitanden mittels nichtstandardisiertem Fragebogen durch. Beurteilt wurden im Vergleich zu den Voraufenthalten die Qualität der ärztlichen Betreuung, der Sprach- und Musiktherapie, der technischen Anpassung und der psychologischen Betreuung sowie der Einsatz der Hygienemaßnahmen.

**Ergebnisse:**

Insgesamt 109 Rehabilitanden beantworteten den Fragebogen. Die Qualität der Rehabilitation und der Therapien wurde als qualitativ unverändert oder besser eingeschätzt. Die Gefährlichkeit der Pandemie, aber auch die Angst in der derzeitigen Situation gaben die Rehabilitanden zu einem unerwartet hohen Prozentsatz mit 68 bzw. 50 % an. Gleichzeitig konnten die getroffenen Hygienemaßnahmen die Patienten subjektiv während des Aufenthalts entlasten. Der Mund-Nasen-Schutz war für die Mehrheit sehr störend, Visiere, Spuckschutz bzw. Abstandsgebot wurden eher toleriert.

**Schlussfolgerungen:**

Die Umsetzung der Hygienemaßnahmen im therapeutischen Setting der CI-Rehabilitation wird von den Rehabilitanden akzeptiert und erlaubt den Zugang zur auditorischen Rehabilitation. Ziel einer erfolgreichen CI-Rehabilitation sollte eine möglichst angstfreie Behandlung unter Wahrung der Hygieneregeln sein.

**Zusatzmaterial online:**

Die Online-Version dieses Beitrags (10.1007/s00106-020-00922-0) enthält den Studienfragebogen. Beitrag und Zusatzmaterial stehen Ihnen auf www.springermedizin.de zur Verfügung. Bitte geben Sie dort den Beitragstitel in die Suche ein, das Zusatzmaterial finden Sie beim Beitrag unter „Ergänzende Inhalte“.

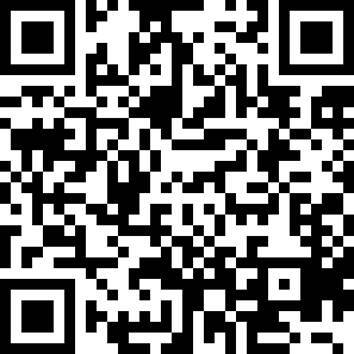

## Abstract (en)

**Background:**

The rehabilitation process following cochlear implant (CI) surgery is carried out in a multimodal therapy according to German national guidelines and includes technical and medical aftercare. In times of the corona pandemic surgery and rehabilitation appointments were cancelled or delayed leading to a more difficult access to auditory rehabilitation. Newly implemented hygiene modalities due to the SARS-CoV‑2 pandemic have changed medical aftercare and the rehabilitation process. The aim of this study was to evaluate the quality of rehabilitation under corona conditions.

**Material and methods:**

An anonymous survey of adult cochlear implant patients was carried out by a non-standardized questionnaire. Demographics were analyzed and the quality of medical aftercare, speech therapy, technical aftercare, psychological support and the hygiene modalities were compared to previous rehabilitation stays.

**Results:**

In total 109 patients completed the questionnaire. The quality of rehabilitation and individual therapy were rated as qualitatively similar or improved. The threat of the pandemic and fear of corona were rated unexpectedly high with 68% and 50%, respectively. The hygiene measures during the rehabilitation stay eased subjective fears at the same time. The majority of patients were annoyed by wearing face masks but visors, protection shields and social distancing were more tolerated.

**Conclusion:**

The implementation of the new hygiene modalities within the therapeutic rehabilitation setting was well-accepted by patients allowing access to auditory rehabilitation. A successful rehabilitation should ensure a fear-free environment by adhering to the necessary hygiene modalities.

## Einleitung

Die Corona-Pandemie hat die Arbeitsabläufe in Kliniken, Praxen aber auch Rehabilitationskliniken massiv verändert. Mit Beginn des sog. Lockdown nach dem 17. März 2020 wurden Ambulanzen reduziert, Operationen abgesagt und verschoben, auch Rehabilitationen fanden nicht mehr oder nur sehr eingeschränkt statt. Durch das Absetzen des sog. Elektivprogramms wurden Intensivkapazitäten freigesetzt, um mit dem SARS-CoV-2(„severe acute respiratory syndrome coronavirus 2“) infizierte Patienten unter besonderen Hygienemaßnahmen behandeln zu können. Schätzungen gehen davon aus, dass im HNO(Hals-Nasen-Ohren)-Bereich bis zu 38,9 % der chirurgischen Eingriffe bei malignen Erkrankungen und bis zu 81,5 % bei nichtmalignen Erkrankungen abgesetzt und/oder verschoben wurden [1]. Verschiebungen dieses Ausmaßes haben eine erhebliche Bedeutung für die Patienten und das Gesundheitssystem. Selbst bei einer anschließenden Erhöhung der operativen Kapazitäten um 20 % könnte es etwa 40 Wochen dauern, diesen Operationsüberhang abzubauen [1].

Die Auswirkungen insbesondere auf schwerhörige Patienten sind nicht bekannt. Allerdings wurden in den meisten Kliniken auch CI(Cochleaimplantat)-Operationen abgesetzt und verschoben. Analoges ist für die Rehabilitation nach CI anzunehmen, da mindestens die Folgetherapie für ca. 2 Monate komplett gestoppt wurde. Das bedeutet, dass für schwerhörige Patienten der Zugang zur auditorischen Rehabilitation zumindest verzögert wurde.

Zwei Monate später, mit der schrittweisen Lockerung der Hygienemaßnahmen, wurden operative Kapazitäten hochgefahren und die Rehabilitationskliniken nahmen, wenn auch eingeschränkt, ihre Arbeit wieder auf. Dabei galten grundsätzlich besondere Hygienemaßnahmen, Abstandsregelungen und Maskenpflicht sowie eingeschränkte Besuchsregelungen und dadurch bedingt eine zahlenmäßige Reduktion der Kapazitäten.

Diese Situation stellt sich als eine besondere Herausforderung für die Rehabilitation nach CI-Versorgung dar. Die Rehabilitation ist laut Leitlinie integraler Bestandteil der CI-Versorgung [7]. Die interdisziplinäre Versorgung umfasst: die medizinische Betreuung, technische Kontrollen, die schrittweise Optimierung der CI-Prozessoreinstellung, die intensive Hör-Sprach-Therapie, die logopädische, phoniatrische, pädagogische und psychologische Diagnostik, die audiometrischen Hör- und Sprachtests (in Ruhe und im Störgeräusch), die Beratung des Patienten und seines sozialen Umfeldes, die psychologische Betreuung, die weitere Schulung in der Handhabung des CI-Systems (Pflege, Wartung, Fehlererkennung) und in der Nutzung von Zusatzgeräten, die Dokumentation und Evaluation der Ergebnisse im Rahmen der wöchentlichen Teambesprechungen sowie die Beratung durch den Sozialdienst zum Behindertenrecht und beruflicher Integration. All dies erfolgt, um Inklusion und Teilhabe entsprechend der International Classification of Functioning, Disability and Health der WHO (World Health Organization) zu ermöglichen.

Die nach Q‑Reha zertifizierte CI-Rehabilitation am Implant Centrum Freiburg (ICF) erfolgt für Erwachsene für 20 Rehabilitationstage als Intervallrehabilitation. Dabei schließen sich 2‑ oder 3‑tägige Rehabilitationsaufenthalte im Verlauf von 24 Monaten der 5‑tägigen Basistherapie an. Bedingt durch die Corona-Pandemie wurde die Rehabilitation in reduzierter Patientenzahl Anfang Mai 2020 wieder gestartet. Die Rehabilitanden und ihre Begleitpersonen erhielten bei Aufnahme direkt eine ärztliche Untersuchung einschließlich Temperaturmessung und Information über Hygienemaßnahmen. Der übliche Terminplan wurde entsprechend der Hygienevorgaben modifiziert, da Gruppentherapien oder -gespräche nicht möglich waren. Technische Anpassungen, logopädische Diagnostik und Therapie, psychologische Gespräche, Musiktherapie und Beratungen erforderten den Einsatz von Mund-Nasen-Schutz (MNS), Visieren, Spuckschutz und die Einhaltung des Abstandsgebots. Die Belegung des Speiseraumes wurde reduziert und die Mahlzeiten in Schichten eingenommen. Durch die Reduktion der Anzahl von Rehabilitanden auf ca. 50 % im Vergleich zur Vor-Corona-Zeit ergaben sich für die Patienten weniger Kontaktmöglichkeiten zum persönlichen Austausch.

Mit der vorliegenden Untersuchung soll untersucht werden, inwieweit die derzeitigen Veränderungen durch Anwendung der aktuellen Hygienemaßnahmen, Abstandsregelungen und Maskenpflicht den Ablauf und den subjektiven Erfolg der CI-Rehabilitation im Vergleich zu Vor-Corona-Zeiten beeinflussen. Darüber hinaus stellt sich die Frage, wie CI-Patienten die Pandemie einschätzen. Untersuchungen zur Rehabilitation nach CI unter COVID(„corona virus disease“)-19-Bedingungen liegen bisher nicht vor.

## Material und Methoden

Wir führten eine anonyme Patientenbefragung im Zeitraum vom 13. Mai 2020 bis zum 25. Juni 2020 durch. Eingeschlossen wurden erwachsene Patienten, die sich zum zweiten Mal oder bereits mehrfach zur Rehabilitation nach CI am ICF vorstellten.

Alle in der stationären Rehabilitation geplanten Patienten wurden im Rahmen der Triage 3 –5 Tage vor Aufnahme telefonisch kontaktiert und zum Gesundheitsstatus sowie zu möglichen Kontakten zu COVID19-Erkrankten befragt. Dabei wurde anonym evaluiert, wie viele Patienten ihren geplanten Termin absagten bzw. verschieben wollten.

Die Hygienemaßnahmen wurden in enger Abstimmung und Beratung mit dem Institut für Krankenhaushygiene festgelegt. Priorität hatten die Distanzregelung und der MNS. Visier und Spuckschutz kamen in den Situationen zum Einsatz, in denen Distanz oder MNS aus medizinisch/therapeutischen Gründen nicht möglich war. Entsprechend den klinikweiten Vorgaben wurde auf Händedesinfektion Wert gelegt.

Die Patienten, die sich zur Rehabilitation vorstellten, wurden im Rahmen der Aufnahmeuntersuchung über die Studie informiert und erhielten die schriftliche Patienteninformation und den Fragebogen (elektronisches Zusatzmaterial online). Dieser wurde anonym am Ende des 2‑ bis 3‑tägigen Aufenthalts abgegeben.

Der nichtstandardisierte Fragebogen enthielt insgesamt 44 Fragen zu medizinischen, psychologischen, therapeutischen und technischen Bereichen. Dabei wurde der qualitative und quantitative Vergleich der Rehabilitation vor und unter Corona-Bedingungen abgefragt. Die empfundene Angst vor der Corona-Pandemie, die Qualität der Rehabilitation und die Zielerreichung unter Pandemie-Bedingungen wurden erfragt. Der Einsatz der verschiedenen Hygienemaßnahmen, MNS, Visiere, Spuckschutz und Abstand, wurde bewertet. Die Bedeutung des Austauschs zwischen den Rehabilitanden sowie die Reduktion der Kontakte durch die Hygienemaßnahmen wurden evaluiert. Ebenso wurden die Zugehörigkeit zu Altersgruppen (18–29 Jahre, 30–39 Jahre usw.), die subjektive Einschätzung der Zugehörigkeit zur Risikogruppe, die Rehabilitationserfahrung, die Mitaufnahme einer Begleitperson und das Geschlecht erhoben.

Die Auswertung erfolgte deskriptiv.

Diese Studie wurde unter der Nr. 00021680 beim Deutschen Register Klinischer Studien registriert.

Mit Antrags-Nr. 10020/20 der Ethik-Kommission der Universität Freiburg erfolgte die Freistellung von der Beratungspflicht, da nur anonymisierte Daten erfasst und ausgewertet wurden.

## Ergebnisse

Im Zeitraum vom 13. Mai 2020 bis zum 25. Juni 2020 wurden 129 Fragebögen an die Rehabilitanden ausgegeben. Die Rücklaufquote betrug 84,5 % (*n* = 109).

Ergebnis der Triage: 48,1 % (*n* = 120 von geplanten 249 Rehapatienten) der erwachsenen Patienten sagten im Rahmen der telefonischen Vorevaluation ihren Termin ab. Die Verteilung der Absagen über die Kalenderwochen 17–26 war konstant.

**Figure 1:**
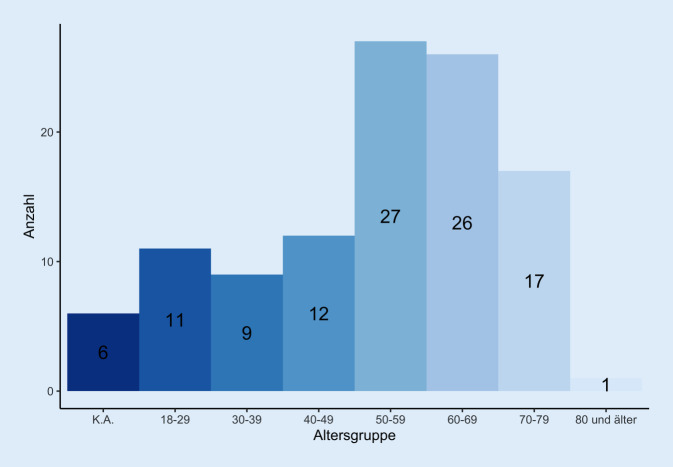

**Figure 2:**
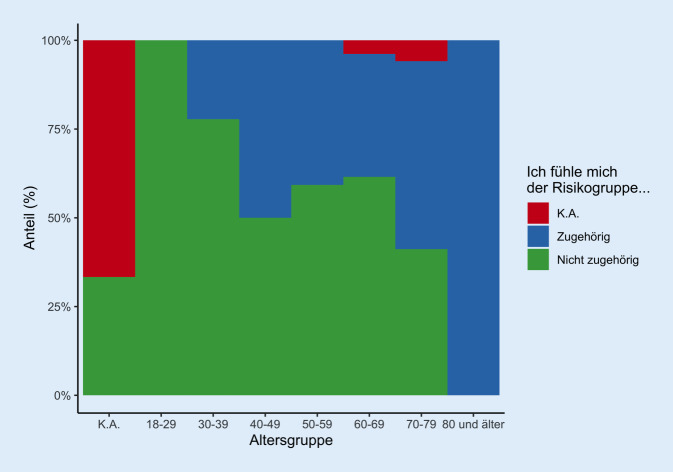

Die Altersverteilung der der Rehabilitanden (*n* = 60 Männer, *n* = 43 Frauen, *n* = 6 keine Angabe) ist in Abb. 1 aufgeführt. Die Patienten kamen zu 47,6 % mit einer Begleitperson. Insgesamt gaben 63,1 % an, sich der Risikogruppe zugehörig zu fühlen. In den verschiedenen Altersgruppen lag die Einschätzung als „zur Risikogruppe zugehörig“ bei zwischen 0 und 100 %: Junge Patienten fühlten sich nicht und 1 betagter Patient (80+ Jahre) als der Risikogruppe zugehörig (Abb. 2), ältere Patienten (70–79 J.) fühlen sich noch zu 40 % als nicht zur Risikogruppe zugehörig.

Die Mehrheit der Patienten beurteilte die Aufnahmeuntersuchung positiv: Der aufnehmende Arzt war verständnisvoll (86,1 %), über Hygienemaßnahmen wurde aufgeklärt (89,8 %) und ein MNS wurde ausgehändigt (95,3 %).

Ein psychologisches Erstgespräch findet regelhaft nur während der Basistherapie statt. Während der Reha wünschten und erhielten 18,3 % (*n* = 19) der Patienten ein erneutes Gespräch. Eine Diskussion über Corona wurde mehrheitlich nicht gewünscht (15 von 19 Patienten). Insgesamt konnten Anregungen für alltägliche Probleme gegeben werden (13 von 15 Patienten), die Rehabilitanden waren durch die Gespräche eher entlastet (11 von 18 Patienten) und Zusammenhänge konnten verdeutlicht werden (14 von 18 Patienten).

Logopädische Therapie und technische Einstellungen der Sprachprozessoren erfolgten bei allen Rehabilitanden und wurden mit gleichbleibender Qualität oder sogar besser als vor Corona bewertet (Logopädie 94,3 %, Technik 97,1 %). Die Musiktherapie wurde mit gleichbleibender Qualität beurteilt, allerdings erhielten nur 44,3 % der Patienten diese Therapie durch die Einschränkung der Gruppentherapien.

Während 86,9 % der Rehabilitanden die Intensität der Therapien im Vergleich zur Vor-Corona-Zeit als genau richtig beurteilten, gaben immerhin 11,2 % eine zu geringe Intensität an. Ihre Therapieziele konnten 89,6 % der Patienten erreichen oder weitgehend erreichen. Insgesamt 10,4 % konnten dies nicht oder nur eingeschränkt.

In der Einschätzung, ob das ICF die richtigen Therapien für das jeweilige Problem vorhält, bewerteten 82,4 % dies positiv; nur 3 Patienten (2,5 %) waren der Meinung, dass das ICF nicht oder eher nicht die richtigen Therapien anbietet.

**Figure 3:**
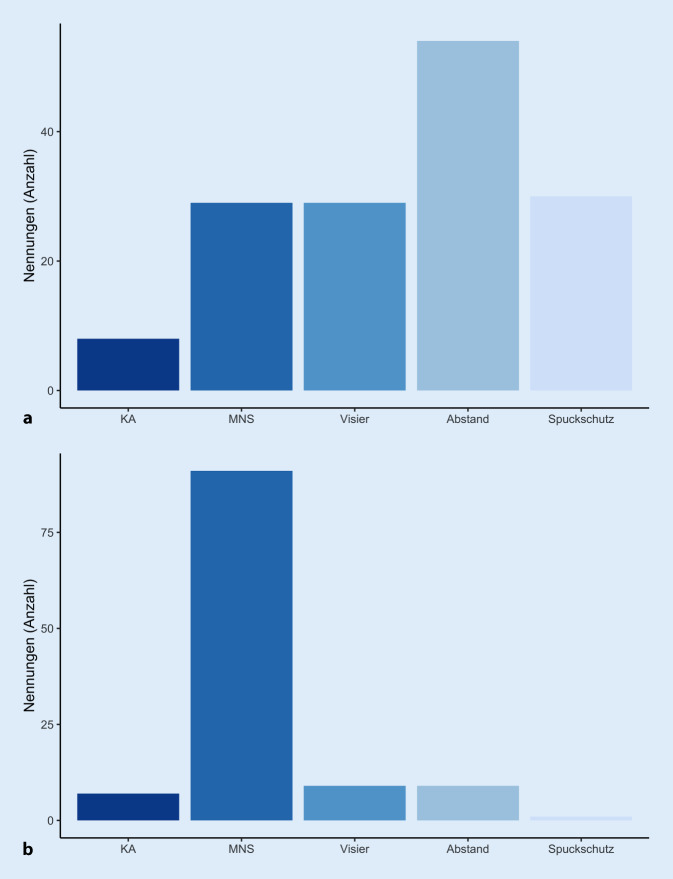

Der Einsatz des MNS wurde mehrheitlich (65,1 %) als störend empfunden, 48 % der Patienten bewerteten die Behandlung dadurch als erschwert. Nahezu die Hälfte der Patienten (43,6 %) empfand die eigene persönliche Schutzausrüstung (MNS) als störend, 25,5 % die der Therapeuten und 30,9 % die von beiden. Die Bewertung der einzelnen Schutzmaßnahmen zeigte, dass die Abstandsregelung im Vergleich zu Visier, Spuckschutz und MNS als am wenigsten störend für die Therapie empfunden wurde und der MNS als am meisten störend (Abb. 3).

Die Mehrheit der Rehabilitanden (55,3 %) hielt die Gespräche mit anderen Rehabilitanden für wichtig oder sehr wichtig. Die getroffenen Schutzmaßnahmen beeinträchtigen diese Gespräche mäßig bis stark für 50 % der Patienten.

**Figure 4:**
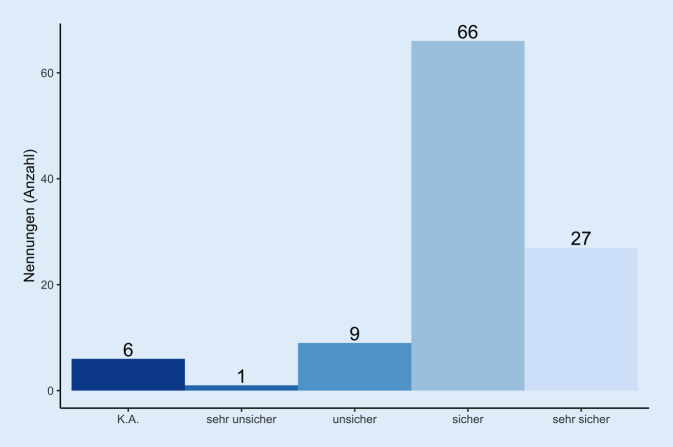

Für die große Mehrheit der Rehabilitanden (89,3 %, Abb. 4) erhöhten die getroffenen Hygienemaßnahmen die empfundene Sicherheit, wobei 68 % die Pandemie als gefährlich und 9 % die Pandemie als harmlos einschätzen.

Die befragten Rehabilitanden gaben demgegenüber Angst vor Corona allgemein zu 50 % an und dies war gleichmäßig verteilt über die Altersstufen. In der Rehaklinik, also während des Rehaaufenthalts, reduzierte sich dieses Empfinden, sodass 81,5 % wenig oder überhaupt keine Angst empfanden. Dies korrelierte mit der Angabe zu „keine Angst vor Corona im Moment“ mit 75 %; eine subjektive Verstärkung des Angstgefühls in der Rehaklinik gaben 18,4 % und „im Moment“ 25 % an.

## Diskussion

Die Notwendigkeit einer postoperativen Rehabilitation nach CI-Versorgung ist in Deutschland anerkannt und im Weißbuch und in der Leitlinie beschrieben [7, 8]. Es liegen verschiedene ambulante und stationäre Konzepte vor, die eine Intervall- oder Blockrehabilitation nutzen [12, 17]. Allen gemeinsam ist der interdisziplinäre Ansatz der Therapie um eine bestmögliche Teilhabe und Inklusion nach WHO zu erreichen. Unter Pandemiebedingungen ist davon auszugehen, dass für Schwerhörige der Zugang zur auditorischen Rehabilitation erschwert ist. Dies ist bedingt durch die Reduktionen der Operationen ebenso wie durch den Stopp der Rehabilitation oder Einschränkungen der technischen Nachsorge.

Die vorgelegte Untersuchung beschreibt die subjektiven Auswirkungen der nach Wiedereinsetzen der Rehabilitation getroffenen Maßnahmen für die Rehabilitanden. Die Rücklaufquote von 84,5 % der anonymen Befragung ist als extrem positiv zu bewerten und drückt vermutlich einerseits ein hohes Interesse der Rehabilitanden an der Befragung aus, aber auch den Wunsch, eine Bewertung des Rehabilitationsprozesses abzugeben. Durch die hohe Rückläuferzahl konnte die Befragung innerhalb kurzer Zeit abgeschlossen werden, sodass ein Gewöhnungseffekt auf die getroffenen Maßnahmen nicht wahrscheinlich ist. Die im Zeitverlauf der Befragung konstante Quote an abgesagten Rehabilitationen im Rahmen der telefonischen Vor-Evaluation spricht ebenfalls gegen einen Gewöhnungseffekt.

Die Erläuterungen zu den Hygienemaßnahmen von medizinischer Seite wurden als positiv empfunden und sicherten den Rehabilitationsaufenthalt.

Dies gilt allerdings bei reduzierter Patientenzahl und bedeutet einen weiter bestehenden Patientenüberhang ohne Abbau. Ein weiteres Problem stellen die Absagen von Patienten dar, die aus Sorge vor einer Infektion während des Aufenthalts oder der Reise (z. B. Zugfahrt) ihren Rehaaufenthalt „auf später“ verschieben. Dadurch entsteht weiterer Patientenüberhang. Darüber hinaus besteht bei Verschiebungen im Rahmen einer Intervallrehabilitation, sei es stationär oder ambulant, immer auch das Risiko, dass durch zu große Abstände zwischen den einzelnen Therapien und Einstellungen nicht das volle Potenzial nach CI-Versorgung erreicht wird oder zeitweise eine verbesserungswürdige Programmierung mit eingeschränktem Sprachverstehen besteht. Auch für taube und schwerhörige Patienten gilt, dass chronische Krankheiten keine „Corona-Pause“ machen [13].

Der Einsatz der Hygienemaßnahmen erschwert die bisher gewohnte Rehabilitation. Dabei wurde der MNS sowohl für die Patienten als auch für die Behandlung als am meisten störend empfunden. In 2020 publizierte Untersuchungen von Saile und Gregori [6] zeigten eine Dämmwirkung von MNS von bis zu 10 dB. Dies stellt für Schwerhörige, die darüber hinaus eventuell auf das Mundbild angewiesen sind, eine massive Einschränkung der Kommunikation dar, wie es sich in den Befragungsergebnissen wiederspiegelt. Der Einsatz von einem transparenten MNS oder von Visieren mag hier Abhilfe schaffen, wobei in unserer Untersuchung die Visiere ebenso wie der Spuckschutz im Vergleich tatsächlich besser abschneiden. Dies ist für die Betreuung Schwerhöriger in der Praxis zu bedenken. Mindestens ein Visier sollte griffbereit sein, damit eine sinnvolle Kommunikation zwischen Arzt oder Therapeut und Patient möglich ist. Die Dämpfung durch MNS usw. sollte bei der Programmierung bedacht werden. Vorstellbar sind dämpfungs- und frequenzspezifische Programme für eine Maskensituation und Programme für Ohne-Maske-Situation, z. B. im privaten Umfeld. Auf den Einsatz und die Problematik des MNS weisen zum Beispiel Deutscher Schwerhörigenbund e. V. und der Landesverband der Gehörlosen Baden-Württemberg auf ihren Webseiten hin [9, 10].

Interessant ist, dass die Rehabilitanden zwar die Einschränkungen durch die Hygienemaßnahmen durchaus negativ bemerkten, die Qualität der Rehabilitation jedoch unverändert gut oder sogar besser beurteilten. Dies gilt für alle Bereiche der Rehabilitation, wie Logopädie, Technik und Musiktherapie. Das veränderte Setting mit MNS, Visier, Spuckschutz usw. hatte keinen negativen Effekt auf den Rehabilitationserfolg und die Zielerreichung. Dies kann einerseits Ausdruck der Akzeptanz der getroffenen Maßnahmen durch die Patienten sein, aber auch der erfolgreichen und einfühlsamen Umsetzung dieser Maßnahmen durch Mediziner, Therapeuten und Techniker.

Angst vor der Corona-Pandemie wird in der Allgemeinbevölkerung mit aktuell ca. 30 % seit Anfang Mai 2020 angegeben [2]. In der vorliegenden Untersuchung findet sich jedoch im Rahmen der Evaluation der erwachsenen Rehabilitanden die Einschätzung der Pandemie als gefährlich, bei 68 und 50 % empfinden Angst vor Corona. Damit ergeben sich deutliche Hinweise, dass Schwerhörige und CI-Patienten die Pandemie anders als die Allgemeinbevölkerung einschätzen. Dies kann durch reale und empfundene Einschränkungen der Kommunikation, der Bewegungsfreiheit und des Zugangs zur Hörrehabilitation bedingt sein und korreliert mit den Ergebnissen der Mannheimer Corona-Studie für Menschen mit eingeschränkter Gesundheit [15]. Interessant ist, dass die Befragten in der Bewertung der empfundenen Angst vor Corona diese während der Reha mehrheitlich als reduziert angaben. Dies kann als Entlastung innerhalb der Kliniksituation gedeutet werden und zeigt vermutlich die positive Bedeutung der getroffenen Hygienemaßnahmen und der veränderten ärztlichen Beratung bei Aufnahme. Damit einher geht auch der geringe Anteil von Patienten (nur 18,3 %), die eine psychologische Betreuung wünschten und praktisch nicht (78,9 %) an einem Gespräch über Corona interessiert waren.

Darüber hinaus haben wir nur Patienten befragt, die sich am ICF zur Therapie befanden und die nicht aus Sorge vor der Pandemie ihren Aufenthalt abgesagt oder verschoben haben. Dies stellt eine Limitation der vorgelegten Untersuchung dar. Diese Beschränkung bedeutet aber auch, dass Schwerhörige oder CI-Patienten möglicherweise zu einem noch viel höheren Prozentsatz Einschränkungen und Ängste durch Corona erfahren als die Normalbevölkerung, insbesondere, da zu postulieren ist, dass besonders ängstliche Patienten Rehatermine eher absagen. Zur weiteren Untersuchung sind strukturierte Interviews in diesem Patientenkollektiv sinnvoll, um den Umgang mit Ängsten für schwerhörige Patienten zu Pandemiezeiten zu optimieren.

Eine weitere Limitation unserer Studie ist die Verwendung eines nichtstandardisierten Fragebogens. Dieser wurde aufgrund der komplett neuen Situation einer weltweiten Pandemie, der zeitlichen Relevanz des Themas und der Ermangelung eines validierten Fragebogens erstellt.

Die Ergebnisse unserer Befragung sind eine Bestätigung des Hygienekonzeptes des ICF. Dieses Konzept betrifft nicht nur die Patienten, sondern muss auch den Mitarbeitern ein Gefühl von Fürsorge durch die Leitung und bestmögliche Sicherheit bei den Arbeitsabläufen im Umgang mit den Patienten bieten. Dies führt zu einer trotz Corona ruhigen und besonnenen Arbeitsatmosphäre. Diese musste sich das Team des ICF erarbeiten. Wesentlich dazu beigetragen hat die wöchentliche Telefonkonferenz aller leitenden Mitarbeiter einschließlich der Qualitätsmanagementbeauftragten (QMBs)unter Führung der Sektionsleitung und die an alle Mitarbeiter wöchentlich gesendeten Protokolle. Auch dies ist indirekt durch die Patienten evaluiert worden und zwar positiv, da die Gefährdung durch Corona eher hoch, aber die Ansteckungsgefahr am ICF eher gering bewertet wurde.

## Ausblick

Die derzeitige Pandemiesituation führt zu Einschränkungen für schwerhörige Patienten. Der Zugang zur auditorischen Rehabilitation erscheint erschwert. Trotz aller bisherigen Lockerungen sagt immer noch ein hoher Prozentsatz der Patienten geplante Rehatermine ab. Dies kann dazu führen, dass Patienten unzureichende Einstellungen ihrer Sprachprozessoren über längere Zeit nutzen und nicht in die Lage versetzt werden, ihr volles Hörpotenzial zu erreichen; dies widerspricht dem Ziel der Teilhabe.

Der Einsatz elektronischer Medien zur Rehabilitation ist in Deutschland zwar im Einzelfall zur Unterstützung der Rehabilitation gebräuchlich (Lern-Apps etc.), könnte jedoch zukünftig eine weitere Therapieform darstellen oder existierende Konzepte ergänzen. Es liegen zwar Berichte über Remote-Mapping vor [14], allerdings wird es im deutschsprachigen Raum wenig eingesetzt. Im angloamerikanischen Raum, im dem eine Rehabilitation in der uns geläufigen Form nicht existiert [3], werden grundsätzlich positive Effekte eines Online-Auditory-Trainings beschrieben [4, 5, 16]. Es stellt sich jedoch die Frage, ob diese Trainingsformen alleine (Mapping plus Auditory-Training) den Begriff der „Rehabilitation“ nach WHO erfüllen [12]. Auch ist für den deutschsprachigen Raum die Finanzierung derartiger Modelle bisher nicht geklärt. Grundsätzlich ist in Analogie zum Home-Office der vermehrte Einsatz elektronischer Medien in Kombination mit klassischer Rehabilitation vor Ort denkbar um Hörpotenziale für die Patienten zu heben [11]. Weitere Evaluationen zur Online-Rehabilitation sind jedoch notwendig, um Effizienz und Effektivität zu testen, aber auch, um die Grenzen aufzuzeigen. Der Kontakt zwischen Rehabilitanden ist zur Sicherung und Optimierung der Rehabilitation nicht zu unterschätzen. Die Mehrheit der befragten Patienten empfindet diese Kontakte als wichtig und bemängelte die Einschränkungen dieser sozialen Kontakte durch die notwendigen Hygienemaßnahmen und die reduzierte Patientenanzahl.

Das Krankenausentlastungsgesetz sieht eine finanzielle Unterstützung von Krankenhäusern und Rehakliniken während der Corona-Pandemie vor. Diese Unterstützung wird jedoch aus eigener Erfahrung die Verluste nicht auffangen können. Die derzeitig notwendige Reduktion der Patientenanzahl und die Veränderungen der Rehabilitation aufgrund der Hygienemaßnahmen (z. B. keine Gruppenangebote oder reduzierte Patientenanzahl bei Gruppenschulungen oder -therapien, zeitintensivere Vor- und Nachbereitung von Therapieräumen) führen zu einer zusätzlichen Aufblähung der Wartelisten und werden auf Dauer die Rehabilitation verteuern. Dies sollte bei zukünftigen Verhandlungen mit den Kostenträgern bedacht werden.

## Fazit

Eine erfolgreiche, relativ angstfreie Rehabilitation in COVID(„corona virus disease“)-19-Zeiten ist möglich. Den Rehabilitanden gelingt eine Adaptation an die Gegebenheiten, auch wenn die Hygienemaßnahmen, insbesondere der MNS für die Mehrheit störend ist. Der Gebrauch von Visieren oder transparentem MNS (Mund-Nasen-Schutz) sollte für die erleichterte Kommunikation selbstverständlich sein. Aufgabe aller an der Rehabilitation Beteiligten ist es, auch unter Pandemiebedingungen eine sichere Umgebung für eine optimale auditorische Rehabilitation unter bestmöglichem Schutz für Patienten, aber auch Mitarbeiter zu schaffen.

## Caption Electronic Supplementary Material


