# Supplementary material for: Quality of cochlear implant rehabilitation under COVID-19 conditions. German version
Source: HNO. 2020 Sep 2;68(11):847–53. [Article in German] doi: 10.1007/s00106-020-00922-0 (PMC7466923; doi:10.1007/s00106-020-00922-0)
Supplement: Supplementary file 1 [file 106_2020_922_MOESM1_ESM.pdf]

Fragebogen

zur Qualitätssicherung der Rehabilitation am ICF im Rahmen der Corona-Pandemie

Dieser Fragebogen untersucht die Qualität der Reha. Ziel ist es, die Veränderungen durch die SARS-CoV-2/Corona-Pandemie in der Rehabilitation zu erfassen, wie im beiliegenden Informationsschreiben ausführlich erläutert.

Bitte lesen die Fragen genau durch und kreuzen Sie die Antwort an, die für Sie zutrifft oder am ehesten zutrifft. Wir bitten um Beantwortung aller Fragen.

Bitte den ausgefüllten Fragebogen in den beiliegenden Umschlag legen und in den Briefkasten mit Aufschrift „Umfrage ICF“ einwerfen. Der Briefkasten hängt in der Eingangshalle im Haus III neben dem Büro von Frau König.

Um Ihre Anonymität zu gewährleiten, bitten wir darum, keinen Namen oder Adresse auf dem Umschlag oder Fragebogen zu vermerken.

Vielen Dank!

1) **Ärztliche Betreuung während der Reha**

Die Ärztin oder der Arzt im Implant Centrum war einfühlsam und verständnisvoll

nein ☐ eher nein ☐ teils ja, teils nein ☐ eher ja ☐ ja ☐

Information über Hygienemaßnahmen (z.B. Masken tragen, Händedesinfektion, Abstand halten) wurden verständlich erläutert

nein ☐ eher nein ☐ teils ja, teils nein ☐ eher ja ☐ ja ☐

Mund-Nasen-Masken wurden mir ausgehändigt

nein ☐ eher nein ☐ teils ja, teils nein ☐ eher ja ☐ ja ☐

2) **Psychologische Betreuung während der Reha**

Haben Sie sich bei diesem Aufenthalt einen Termin bei einer unserer Psychologinnen gewünscht?

Ja ☐ Nein ☐

Haben Sie einen Termin bei der Psychologin erhalten?

Ja ☐ Nein ☐

Falls ja, beantworten Sie bitte die folgenden Fragen (sonst weiter mit 3)

Hatten Sie in dem Beratungsgespräch das Bedürfnis, über Ängste und Sorgen aufgrund der aktuellen Corona-Situation zu sprechen?

Ja ☐ Nein ☐

Hat das Gespräch bei der Psychologin Ihnen Anregungen für Ihre alltäglichen Probleme gegeben?

ja ☐ eher ja ☐ teils ja, teils nein ☐ eher nein ☐ nein ☐

Konnte das Gespräch Sie etwas entlasten?

ja ☐ | eher ja ☐ | teils ja, teils nein ☐ | eher nein ☐ | nein ☐

Die Psychologin oder der Psychologe hat mir wichtige Zusammenhänge verständlich erklärt

nein ☐ | eher nein ☐ | teils ja, teils nein ☐ | eher ja ☐ | ja ☐

3) Haben Sie folgende Behandlungen erhalten? Wenn ja, wie beurteilen Sie diese im Vergleich zur Reha vor Corona?

Logopädische Therapie:

Nein, nicht erhalten ☐ | Ja, und sie war im Vergleich ☐

viel besser ☐ | besser ☐ | gleich gut ☐ | schlechter ☐ | viel schlechter ☐

Musiktherapie/Rhythmik

Nein, nicht erhalten ☐ | Ja, und sie war im Vergleich ☐

viel besser ☐ | besser ☐ | gleich gut ☐ | schlechter ☐ | viel schlechter ☐

Technische Anpassung und Beratung:

Nein, nicht erhalten	Ja, und sie war im Vergleich
☐	☐

viel besser	besser	gleich gut	schlechter	viel schlechter
☐	☐	☐	☐	☐

4) **Behandlungen während der Reha**

Die Intensität meiner Betreuung und meiner Behandlungen am ICF waren im Vergleich zur früher

viel zu wenig	zu wenig	genau richtig	zu viel	viel zu viel
☐	☐	☐	☐	☐

Ich konnte unter den veränderten Umständen meine Ziele für diesen Aufenthalt

erreichen	weitgehend erreichen	eingeschränkt erreichen	nicht erreichen
☐	☐	☐	☐

5) **Wie beurteilen Sie die Qualität und Güte der Angebote (Betreuungen, Behandlungen und Beratungen) während der Reha?**

Für meine Beschwerden hatte das ICF die richtigen Betreuungen, Behandlungen und Beratungen

nein	eher nein	teils ja, teils nein	eher ja	ja
☐	☐	☐	☐	☐

Wie beurteilen Sie den Umfang der Angebote (Betreuungen, Behandlungen und Beratungen) im Vergleich zu Ihren vorherigen Aufenthalten vor Corona?

Die Häufigkeit meiner Betreuung und meiner Behandlungen am ICF waren im Vergleich zur Vor-Corona-Zeit

viel zu wenig	zu wenig	genau richtig	zu viel	viel zu viel
☐	☐	☐	☐	☐

6) Persönlicher Schutz

Die persönliche Schutzausrüstung war während der technischen Betreuung, Logopädie, Psychologische Beratung oder Musiktherapie

störend ☐ etwas störend ☐ gar nicht störend ☐ unbedeutend ☐

Die Kommunikation war für mich durch die Persönliche Schutzausrüstung (PSA) erschwert

Ja ☐ Nein ☐

Falls ja:

Besonders störend war für mich…

☐ die PSA des Therapeuten

☐ meine eigene PSA

Am wenigsten fühlte ich mich im Rahmen der Anpassung/Logopädie/Psychologische Beratung und Musiktherapie beeinträchtig durch

Mund-Nasen-Maske ☐ Visier ☐ 2m Abstand ☐ Spuckschutz ☐

Am stärksten fühle ich mich im Rahmen der Behandlungen beeinträchtig durch…

Mund-Nasen-Maske ☐ Visier ☐ 2m Abstand ☐ Spuckschutz ☐

Das Gespräch mit anderen Rehabilitanden außerhalb der Therapie ist für mich…

unwichtig ☐ weniger wichtig ☐ wichtig ☐ sehr wichtig ☐

Die Schutzmaßnahmen (Sitzordnung, Abstand usw.) haben hierauf…

keinen Einfluss ☐ geringen Einfluß ☐ mäßigen Einfluß ☐ starken Einfluß ☐

Durch die Maßnahmen, die im ICF getroffen wurden, fühlte ich mich während des Aufenthaltes…

sehr unsicher ☐ unsicher ☐ sicher ☐ sehr sicher ☐

Die SARS-COV2-Pandemie halte ich für…

gefährlich ☐ harmlos ☐ weiß nicht ☐

7) Wie sehr treffen die folgenden Gefühlsbeschreibungen auf Sie zu?

Wie bedrohlich empfinden Sie für sich die aktuelle Corona-Situation **im Allgemeinen**?

überhaupt nicht ☐ eher wenig ☐ eher mehr ☐ sehr ☐

Wie stark war Ihr Gefühl von Angst aufgrund der aktuellen Corona-Situation **während** Ihres Reha-Aufenthaltes?

überhaupt nicht ☐ eher wenig ☐ eher mehr ☐ sehr ☐

Wie stark ist Ihr Gefühl von Angst aufgrund der aktuellen Corona-Situation **im Moment** bei Ihnen?

überhaupt nicht ☐ eher wenig ☐ eher mehr ☐ sehr ☐

8) Therapieablauf während der Reha

Wann fand die ärztliche Aufnahmeuntersuchung statt?

am Aufnahmetag ☐ am 2. Tag ☐ am 3. Tag ☐

Wann begannen Ihre Behandlungen?

am Aufnahmetag ☐ am 2. Tag ☐ am 3. Tag ☐

Wie häufig fielen während Ihrer gesamten Reha Behandlungen aus?

nie	1-mal	2-mal	3-mal
☐	☐	☐	☐

Im Vergleich zur Reha vor Corona fielen Behandlungen…

häufiger aus	gleich häufig aus	weniger aus
☐	☐	☐

Falls Behandlungen ausgefallen sind: Ich fühlte mich über die Gründe…

gut informiert	wenig informiert	nicht informiert
☐	☐	☐

Ich konnte diese…

nachvollziehen	nicht nachvollziehen
☐	☐

Der Ausfall der Therapien war…

mit Corona verbunden	unabhängig von Corona
☐	☐

9) Unterbringung während der Rehabilitation

Bitte bewerten Sie:

Das Essen im Implant Centrum waren im Vergleich zur Vor-Corona-Zeit…

viel besser	besser	gleich gut	schlechter	viel schlechter
☐	☐	☐	☐	☐

Die Organisation im ICF (z.B. Therapieablauf, Informationen, Terminvereinbarung) war im Vergleich zur Vor-Corona-Zeit …

viel besser	besser	gleich gut	schlechter	viel schlechter
☐	☐	☐	☐	☐

10) **Gesamtbewertung: Zufriedenheit mit der Rehabilitation zur Zeit der Corona-Pandemie**

Wie beurteilen Sie den Erfolg der Reha-Behandlung am ICF im Vergleich zum letzten Aufenthalt vor Corona?

viel besser	besser	teils, teils	schlechter	sehr schlecht
☐	☐	☐	☐	☐

Wie würden Sie Ihre Reha-Maßnahme am Implant Centrum insgesamt im Vergleich zum letzten Aufenthalt vor Corona bewerten?

viel besser	besser	teils, teils	schlechter	sehr schlecht
☐	☐	☐	☐	☐

11) Angaben zur Person

Wie oft waren Sie bereits am Implant Centrum zur Reha?

1-3	4-6	7-9	>9 Aufenthalte
☐	☐	☐	☐

Hatten Sie eine Begleitperson?

Ja	Nein
☐	☐

Sie sind

Männlich	Weiblich
☐	☐

Ihr Alter ist

18-29	30-39	40-49	50-59	60-69	70-79	80 und älter
☐	☐	☐	☐	☐	☐	☐

Ich fühle mich einer Risikogruppe bzgl. Corona

zugehörig	nicht zugehörig
☐	☐
